# Supplementary material for: The creation of electric wind due to the electrohydrodynamic force
Source: Nat Commun. 2018 Jan 25;9:371. doi: 10.1038/s41467-017-02766-9 (PMC5785522; doi:10.1038/s41467-017-02766-9)
Supplement: Supplementary file 1 — Supplementary Information [file 41467_2017_2766_MOESM1_ESM.pdf]

Supplementary Information

Supplementary Note 1: Gas temperature measurements based on optical emission spectroscopy.

The nitrogen molecular ion band N_2^+ $(B^2\Sigma_u^+ - X^2\Sigma_g^+)$ was measured using an Acton Research SpectraPro 750 spectrometer to estimate the gas temperature (see Supplementary Fig. 1a). The excitation of nitrogen occurs when the helium plasma jet interacts with the neighboring air environment. The experimentally measured N_2^+ spectrum and the well-fitted synthetic spectrum with the LIFBASE software [1] presented in Supplementary Fig. 1b indicate a rotational temperature of 340 K. In Supplementary Fig. 1a, the plotted spectra are shifted to facilitate easy comparison.

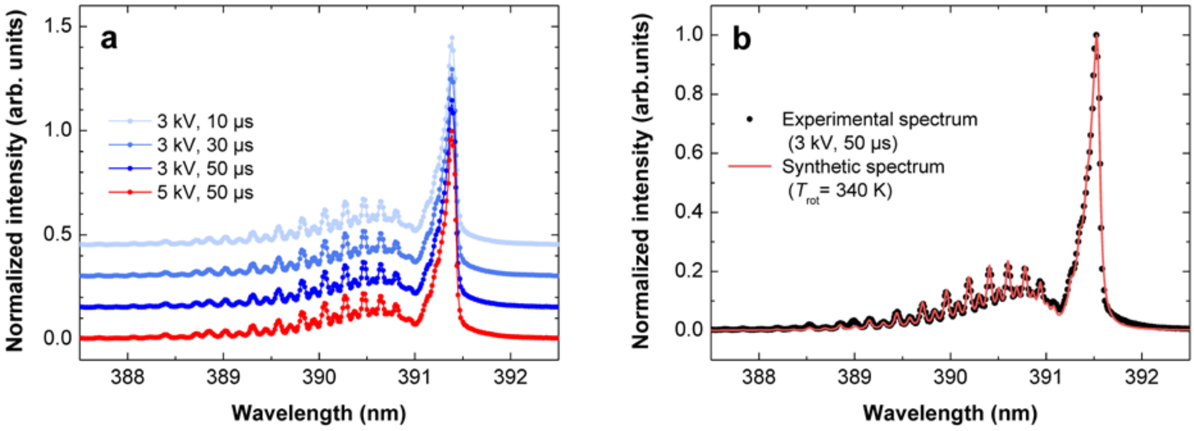

Supplementary Figure 1 | Gas temperature estimation of helium plasma jets under different conditions. (**a**) Rotational distribution of N_2^+ $(B^2\Sigma_u^+ - X^2\Sigma_g^+)$ emission with different pulse widths (10, 30, and 50 μs at 3 kV) and heights (3 and 5 kV at 50 μs) and (**b**) representative experimental and synthetic spectra obtained with T_{rot} = 340 K. The synthetic spectrum of nitrogen molecular emission was simulated using the LIFBASE [1].

Supplementary Note 2: On the background of active species and reaction scheme in the He jet.

Here, we attempt to highlight basic mechanisms and reactions inside the atmospheric pressure helium discharge and at the flow boundary during reactions with the ambient air. There are numerous reactions (Supplementary Table 1) that occur inside the He plasma jet and during interaction with the ambient air. The photo-ionization mechanism based on Dawson's theory frequently plays an important role. In addition, a considerable amount of helium metastables and excited nitrogen molecules exist during the excitation cycle. The electrons are produced by step-wise ionization, super-elastic ionization and metastable pooling of helium metastables as well as through Penning ionization. The channels of electron production become more effective and complex in the continuous mode than in the bullet, or pulsed streamer, mode and can significantly affect the spatial distribution of space charges, which can even overwhelm the contribution of photo-ionization. [2]

When ionized helium gas interacts with the ambient gas, the highly energetic metastables [$He^*(2^1S)$ and $He^*(2^3S)$] are able to cause the ionization of nitrogen molecules. The helium discharge inside the glass tube generates a large flux of long-lived helium metastables (with lifetimes as long as 7870 s [3]); however, due to Penning reactions, the effective lifetimes become much shorter. This leads to a rapid reduction in helium metastables in the interaction zone with ambient air. Here, it is worth noticing that the Penning effect between two nitrogen molecules is crucial in maintaining stable glow discharges, where even $N_4{}^+$ ions are produced from interactions of excited N_2 molecules. Otherwise, the typical ions produced in atmospheric helium plasmas and in contact with nitrogen are He^+, $He_2{}^+$, N^+, $N_2{}^+$, $N_3{}^+$ and $N_4{}^+$, which can be described as positive ions in our descriptions.

Supplementary Table 1 | The most common reactions occurring inside He jet releasing into the ambient air. Crosse-reactions between helium and the ambient air strongly influence the generation and propagation of the pulsed He plasma jets.

No.	Reaction	Rate coefficient	Reference
Helium chemistry			
R1	$He + e \rightarrow He^* + e$	$3.88\times10^{-10}\exp(-1.40\times10^6/E)$	[4]
R2	$He + e \rightarrow He^+ + 2e$	$4.75\times10^{-10}\exp(-2.31\times10^6/E)$	[4]
R3	$He^* + e \rightarrow He^+ + 2e$	$2.02\times10^{-7}\exp(-3.10\times10^5/E)$	[4]
R4	$He_2^+ + e \rightarrow He^* + He$	$8.9\times10^{-9}(T_e/T_g)^{-1.5}$	[5]
R5	$He_2^+ + e \rightarrow 2He$	1.0×10^{-8}	[6]
R6	$He_2^* + e \rightarrow He_2^+ + 2e$	$1.268\times10^{-12}T_e^{0.71}\exp(-3.945\times10^{-4}/T_e)$	[7]
R7	$He^+ + 2He \rightarrow He_2^+ + He$	1.0×10^{-31}	[7]
R8	$He^+ + e \rightarrow He$	2.0×10^{-12}	[6]
R9	$He^* + 2He \rightarrow He_2^* + He$	1.5×10^{-34}	[6]
R10	$He^* + He^* \rightarrow He_2^+ + e$	1.05×10^{-9}	[6]
R11	$He^* + He^* \rightarrow He + He^+ + e$	4.5×10^{-10}	[6]
R12	$He^* + He_2^* \rightarrow He + He + He^+ + e$	5.0×10^{-10}	[6]
R13	$He^* + He_2^* \rightarrow He + He_2^+ + e$	2.0×10^{-9}	[6]
R14	$He_2^* + He_2^* \rightarrow He_2^+ + 2He + e$	1.2×10^{-9}	[6]
R15	$He_2^* + He_2^* \rightarrow He^+ + 3He + e$	3.0×10^{-10}	[6]

Nitrogen chemistry			
R16	$N_2 + e \rightarrow N_2^+ + 2e$	$4.483\times10^{-7}\ T_e^{-0.3}\ \exp(-1.81\times10^5/T_e)$	[7]
R17	$N_2 + e \rightarrow N + N + e$	$1.959\times10^{-6}\ T_e^{-0.7}\ \exp(-1.132\times10^5/T_e)$	[7]
R18	$N_2 + e \rightarrow N_2(C^3\Pi) + e$	$f(T_e)$	[8]
R19	$N_2^+ + e \rightarrow N_2$	$4.8\times10^{-7}\ (T_e/T_g)^{-0.5}$	[10]
R20	$N_2(C^3\Pi) \rightarrow N_2(B^3\Pi) + h\nu$	$2.4\times10^7\ s^{-1}$	[8]
R21	$N_2^+ + 2N_2 \rightarrow N_4^+ + N_2$	1.9×10^{-29}	[10]
R22	$N_4^+ + e \rightarrow N_2 + N_2$	$2.0\times10^{-6}\ (T_g/T_e)^{0.5}$	[10]
R23	$N_4^+ + N_2 \rightarrow N_2^+ + 2N_2$	2.5×10^{-15}	[10]
Helium-nitrogen interactions			
R24	$He^* + N_2 \rightarrow He + N_2^+ + e$	5.0×10^{-11}	[6]
R25	$He^* + N_2 \rightarrow He + N + N^+$	1.0×10^{-20}	[6]
R26	$He_2^* + N_2 \rightarrow 2He + N_2^+ + e$	3.0×10^{-11}	[6]
R27	$He^+ + N_2 \rightarrow He + N_2^+$	6.0×10^{-10}	[6]
R28	$He^+ + N_2 \rightarrow He + N + N^+$	6.0×10^{-10}	[6]
R29	$He_2^+ + N_2 \rightarrow He_2^* + N_2^+$	1.4×10^{-9}	[10]
R30	$He_2^+ + N_2 \rightarrow 2He + N_2^+$	5.0×10^{-16}	[11]
R31	$N_2^+ + N_2 + He \rightarrow N_4^+ + He$	1.9×10^{-29}	[10]
R32	$N_4^+ + He \rightarrow N_2^+ + N_2 + He$	1.0×10^{-10}	[6]

Units: Two-body reaction ($cm^3\ s^{-1}$). Three-body reaction ($cm^6\ s^{-1}$). Electron temperature T_e (K). Gas temperature T_g (K). The electric field E ($V\ m^{-1}$).
Note: $f(T_e)$ is given as a function of electron energy.

Based on previous research on atmospheric pressure plasmas created in jets, there are small differences in voltage and current waveforms (Supplementary Fig. 5) between the positive- and negative-polarity plasma jets (Supplementary Fig. 2). These differences are reasonably small and difficult to observe from only high-resolution optical emission data peaks (Supplementary Fig. 3), where even more sophisticated methods, such as time-resolved laser absorption spectroscopy (LAS) and laser-induced fluorescence (LIF), have to be used for the detection of various species. Typically, the positive polarity leads to the creation of a hollow shape structure of the jet with excited species. In contrast, a negative polarity leads to the creation of a uniformly shaped jet with excited species. [12] Often, it is found that there are high densities (> $10^{11}\ cm^{-3}$) of He metastables elongated along the He flow. However, the ring-shaped distributions, which are attributed to breakdown mechanisms at the electrode, are observed in a positive phase. Accordingly, the absolute densities of He metastables are higher for the negative-polarity discharges at comparable measurement and discharge parameters. In radial profiles of jets, we can find $N_2^+(X)$ ions that tend to diffuse toward the center of the He gas flow, where the quenching of $N_2^+(X)$ is less effective. Meanwhile, on the outer emission edge of the plasma jet, $N_2^+(X)$ ions are quenched by collisions with nitrogen and oxygen molecules, resulting in the production of N_4^+ in O_2^+. Similar phenomena and jet-shaped-emission structure are observed in our case, where Supplementary Fig. 6 highlights images of positive- and negative-polarity jets with a schematic representation of the expected distribution of some major plasma species along the jet flow path.

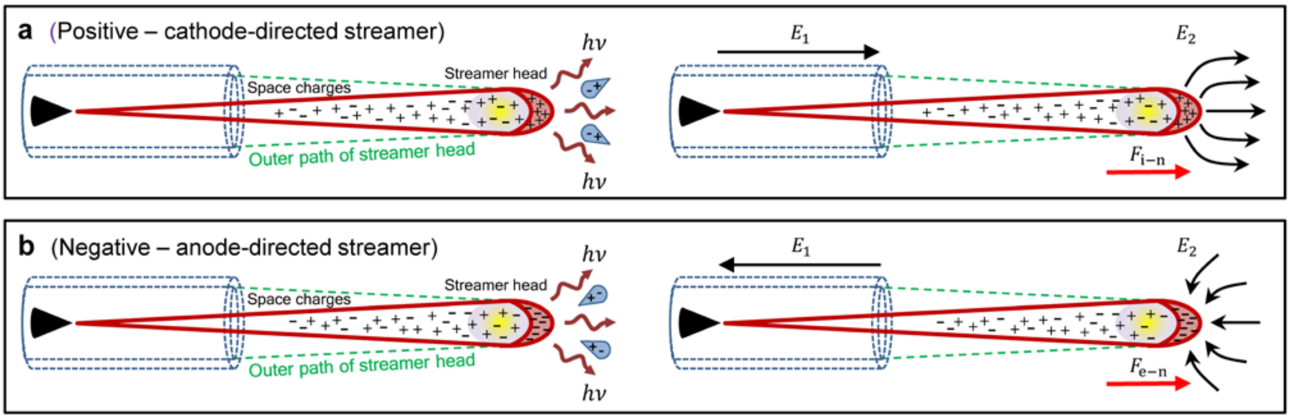

Supplementary Figure 2 | Schematic representation of the moving streamer and space charge. A more detailed explanation of the moving streamer head and tail behind is depicted from images for (**a**) the positive- and (**b**) negative-polarity cases. The left side represents the general properties of the moving streamer head and space charge in the tail in the path of the head from the tube exit, whereas the right side presents the physics of the electric fields. In the case of an anode-directed streamer, electrons are pulled by an electric field into the positively charged tail, creating a rapidly propagating streamer.

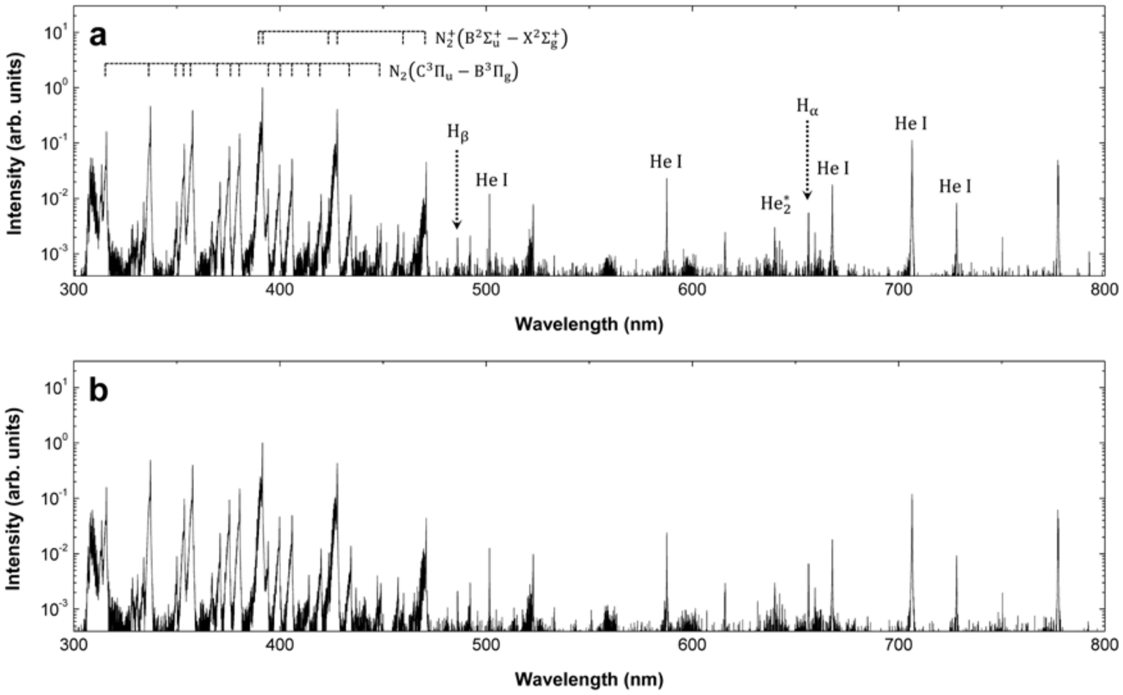

Supplementary Figure 3 | Typical optical emission spectra for the pulsed plasma jets. Log-scaled spectra of (**a**) positive- and (**b**) negative-polarity pulsed plasma jets with a 50 μs pulse width presenting the plasma species emission structure. Both spectra were measured by an Acton Research SpectraPro 750 spectrometer and normalized by maximum intensity (Instrument setting: 10 μm slit width, 0.5 s integration, 5 times averaged, dark spectra subtracted, and structure obtained at the tube exit).

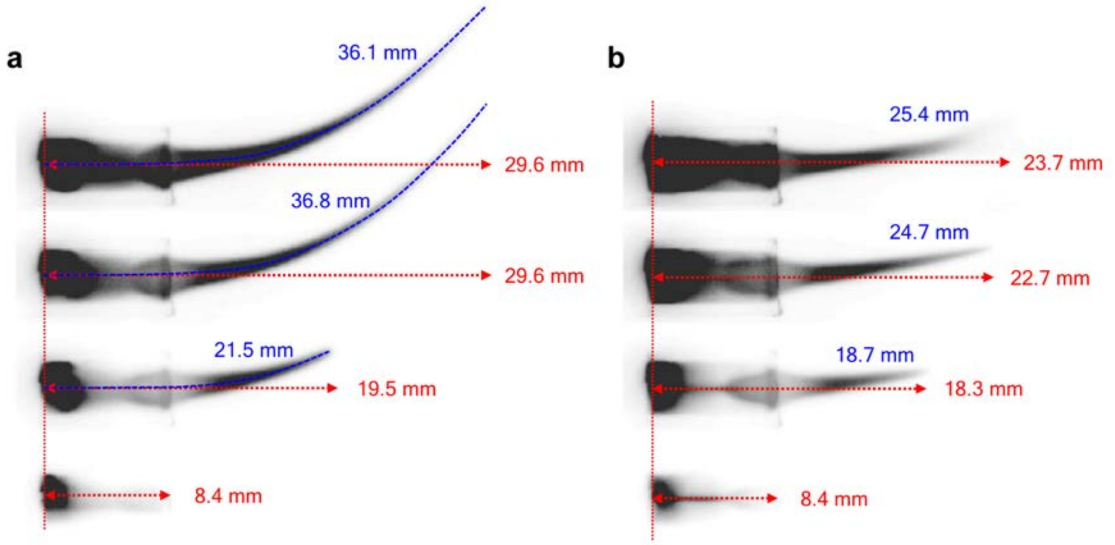

Supplementary Figure 4 | Propagation of the pulsed streamer and the measurements of the jet length. An example of measurements for the time-integrated movement of the created pulsed plasma streamer, from generation to extinction, for two representative cases of discharge parameters (**a**) 1 µs and (**b**) 50 µs at different discharge voltages (5, 4, 3 and 2 kV in order from top to bottom) taken by an Andor DH312T camera. The total jet length is measured along the path of the jet, as depicted by blue dashed lines.

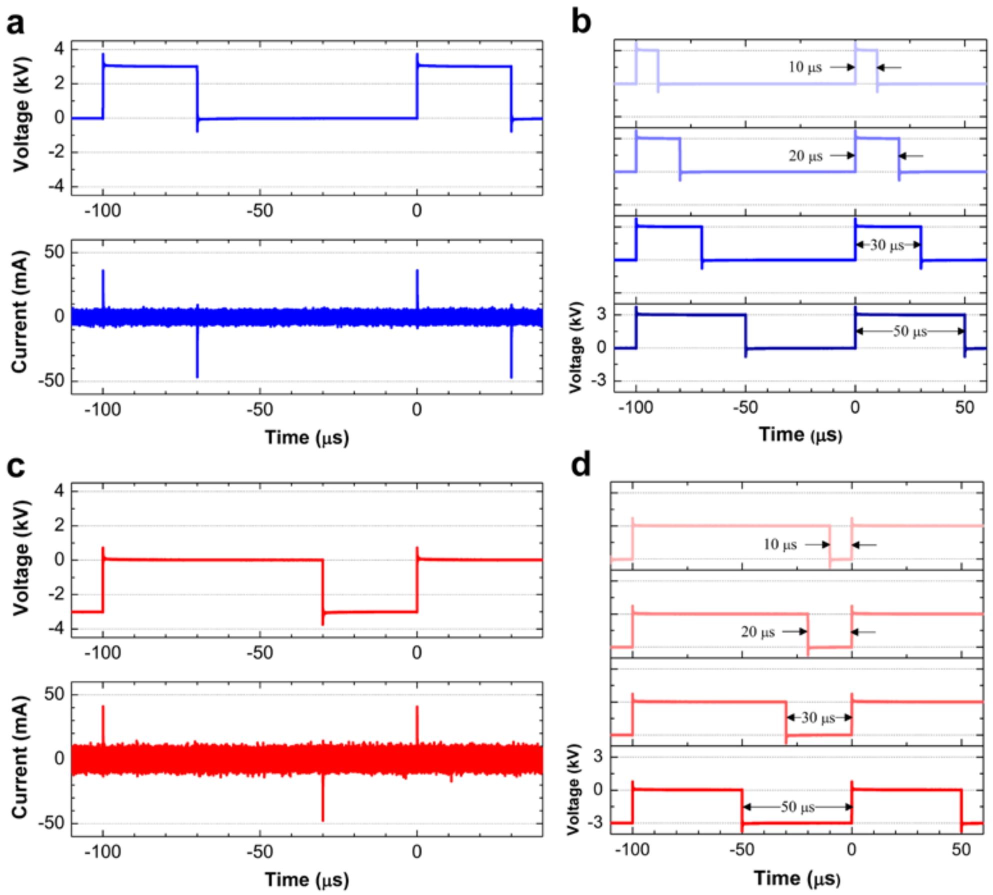

Supplementary Figure 5 | Voltage waveforms of positive- and negative-polarity pulses. (**a**) Measured discharge voltage and discharge current waveforms with a 30-μs pulse width and (**b**) the voltage waveform with pulse widths of 10-50 μs in the positive-polarity pulsed plasma jet. The corresponding figures of the negative-polarity cases are depicted in (**c,d**). Corresponding flow trajectories and electric wind speeds are presented in Fig. 4.

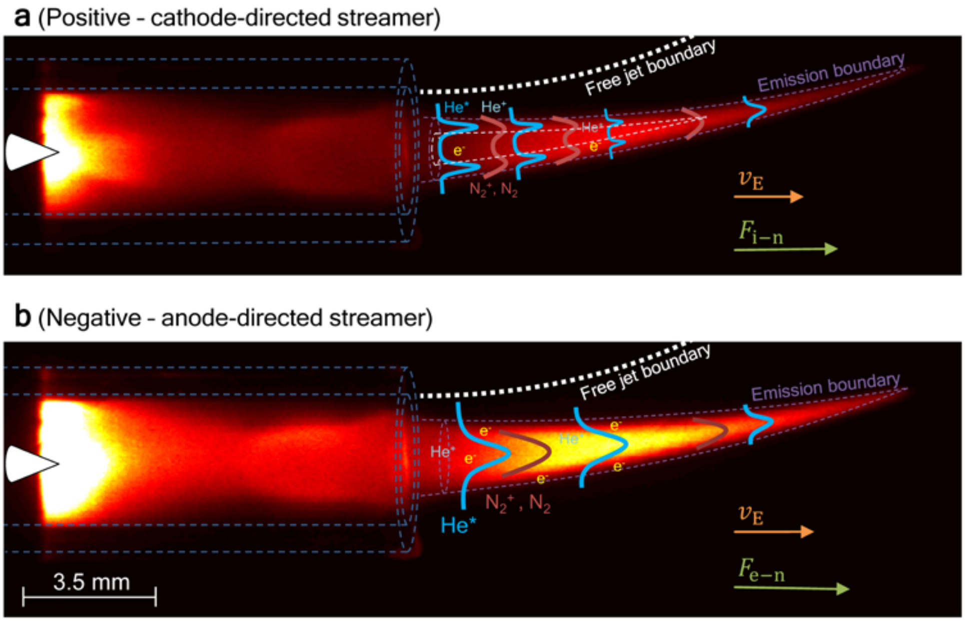

Supplementary Figure 6 | Comparable propagation scheme of the pulsed streamers. The time-integrated sequence of ultra-fast images representing the movement of the created pulsed plasma streamer from generation to extinction for two representative cases of discharge parameters: (**a**) 10 µs at positive 3 kV and (**b**) similarly for negative voltages. The obvious difference is the emission from the jet, which is the result of the hollow shape jet generated with positive polarity and the more uniform shape in the negative polarity discharge. The pictures also present some general features of our experiment and results with a schematic representation of the plasma species distribution in such discharges as observed by other authors [12].

Supplementary Methods

On the model for fitting wind speeds. Because the plasma jets are significantly perturbed by conventional instruments for measuring the flow speed, such as a pitot tube, the experiment for measuring the flow speed of neutral helium in the plasma jet is quite limited. Here, to estimate the electric wind speed, the flow trajectories are analyzed in terms of the gas speed based on the following analytical model [13]:

$$\frac{\mathrm{d}U_\mathrm{c}^{'}}{\mathrm{d}S} = 2\left(A^2\mathrm{Ri}\right)\frac{\phi_\mathrm{c}}{U_\mathrm{c}^{'}}\sin\,\theta - \frac{2\alpha U_\mathrm{c}^{'}}{R^{'}} \tag{1}$$

$$\frac{\mathrm{d}R^{'}}{\mathrm{d}S^{'}} = 2\alpha - \left(A^2\mathrm{Ri}\right)\frac{\phi_\mathrm{c}}{\left(U_\mathrm{c}^{'}\right)^2}\sin\,\theta \tag{2}$$

$$\frac{\mathrm{d}\theta}{\mathrm{d}S^{'}} = 2\left(A^2\mathrm{Ri}\right)\frac{\phi_\mathrm{c}}{\left(U_\mathrm{c}^{'}\right)^2}\cos\,\theta \tag{3}$$

$$\left(R^{'}\right)^2 U_\mathrm{c}^{'}\phi_\mathrm{c} = \mathrm{const.} \tag{4}$$

$$\frac{\mathrm{d}X^{'}}{\mathrm{d}S^{'}} = \cos\,\theta \tag{5}$$

$$\frac{\mathrm{d}Y^{'}}{\mathrm{d}S^{'}} = \sin\,\theta \tag{6}$$

where U_c is the centerline speed of the flow, S is the distance along the jet, θ is the angle of inclination of the jet axis with the horizontal, α is the entrainment coefficient, R is the width of the flow, X is the horizontal distance from the flow exit, and Y is the vertical upward distance from the center of the flow. Some variables, denoted by primes, are made dimensionless by dividing by the tube diameter D. ϕ_c is the dimensionless centerline temperature, defined as $\phi_\mathrm{c} = (t_\mathrm{c} - t_\infty)/(t_\mathrm{i} - t_\infty)$, where t_c is the local temperature measured at a center of the flow, t_i is the measured inlet temperature of the jet, and t_∞ is the ambient air temperature. A is the ratio of the density and velocity field. In the present work, a value of A of 1.0 was chosen, and the variations in A with the discharge along the jet axis are ignored. The parameter α, which is rather insensitive to the flow trajectory [13], was taken as 0.05. A detailed description of the notations and assumptions can be found in [13]. The governing equations were solved numerically via the fourth-order Runge-Kutta method in MATLAB. The governing parameter of this model is the Richardson number Ri, which is a dimensionless parameter that expresses the ratio of potential energy to kinetic energy. The Richardson number is based on the magnitude of the gravitational acceleration g, the inner diameter of the tube D, the average gas velocity V, and the mass densities of air ρ_air and helium ρ_jet:

$$\mathrm{Ri} = gD\left(\rho_\mathrm{air} - \rho_\mathrm{jet}\right)/\,\rho_\mathrm{jet}V^2 \tag{7}$$

The Richardson numbers are calculated using ρ_air= 1.2041 kg·m^{-3} and ρ_jet= 0.164 kg·m^{-3}. As expressed in the equation, the Richardson number is decreased when the gas flow rate is enlarged. All Richardson numbers are less than unity, which indicates that the buoyancy force is insignificant. The starting conditions for governing Eqs. 1-6 are identical to those described in [13]. First, the helium flow trajectory without the plasma jet was compared with a modeled trajectory. As seen in Supplementary Fig. 7a, the trajectory of the pure

helium jet (open circles) is well fitted with a synthetic trajectory (red solid line) of 0.925 slpm. The discrepancy may come from the inaccuracy of the mass flow controller or the above approximated model. All measured trajectories obtained by Schlieren photography were fitted, and corresponding flow speeds were estimated. Supplementary Fig. 7b shows the helium flow trajectories (same data as in Fig. 4a in the main text) and their fitted results (dotted lines) in the presence of a positive-pulsed plasma jet with pulse widths of 10, 20, 30 and 50 μs and a pulse height of 3 kV. The pure electric wind speed (given in Figs. 3f and 4c in the main text) is obtained by subtracting the average gas speed of 1.6 $m \cdot s^{-1}$ corresponding to the supplying gas flow rate of 0.925 slpm from the total gas speed.

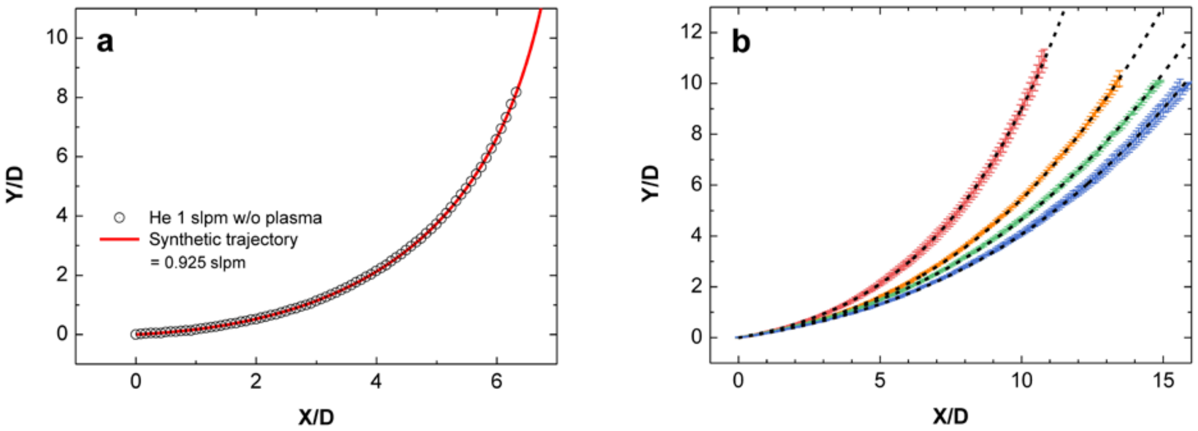

Supplementary Figure 7 | Fitting results of the trajectory modeling for the electric wind speed estimation. (**a**) The measured flow trajectory of the pure helium jet with a 1 slpm flow rate in the experiments (scatters) and the fitted result by the model (red solid line). (**b**) An example of fitted data (black scatters) for helium plasma jet trajectories with the electric wind, which is the same data as in Fig. 4a in the main text.

Supplementary References

1. Luque, J. & Crosley, D. R. Lifbase: Database and spectral simulation program (version 2.1.1). SRI International Report MP 99-009 (1999).
2. Walsh, J. L., Iza, F., Janson, N. B., Law, V. J. & Kong, M. G. Three distinct modes in a cold atmospheric pressure plasma jet. *J. Phys. D: Appl. Phys.* **43**, 075201 (2010).
3. Hodgman, S. S. *et al.* Metastable helium: A new determination of the longest atomic excited-state lifetime. *Phys. Rev. Lett.* **103**, 053002 (2009).
4. Sakiyama, Y. & Graves, D. B. Finite element analysis of an atmospheric pressure RF-excited plasma needle. *J. Phys. D: Appl. Phys.* **39**, 3451-3456 (2006).
5. Smirnov, B. M. *Ions and Excited Atoms in Plasma* (Moscow: Atomizdat, 1974).
6. Tomoyuki, M. *et al.* Chemical kinetics and reactive species in atmospheric pressure helium-oxygen plasma with humid-air impurities. *Plasma Sources Sci. Technol.* **22**, 015003 (2013).
7. Yuan, X. & Raja, L. L. Computational study of capacitively coupled high-pressure glow discharge in helium. *Ieee Trans. Plasma Sci.* **31**, 495-503 (2003).
8. Naidis, G. V. Modelling of plasma bullet propagation along a helium jet in ambient air. *J. Phys. D: Appl. Phys.* **44**, 215203 (2011).
9. Kossyi, I. A., Kostinsky, A. Yu., Matveyev, A. A. & Silakov, V. P. Kinetics scheme of the non-equilibrium discharge in nitrogen-oxygen mixtures. *Plasma Sources Sci. Technol.* **1**. 207-220 (1992).
10. Martens, T., Bogaerts, A., Brok, W. J. M. & Dijk, J. V. The dominant role of impurities in the composition of high pressure noble gas plasmas. *Appl. Phys. Lett.* **92**, 041504 (2008).
11. Breden, D., Miki, K. & Raja, L. L. Self-consistent two-dimensional modeling of cold atmospheric-pressure plasma jets/bullets. *Plasma Sources Sci. Technol.* **21**, 034011 (2012).
12. Urabe, K., Morita T., Tachibana, K. & Ganguly, B. N. Investigation of discharge mechanisms in helium plasma jet at atmospheric pressure by laser spectroscopic measurements. *J. Phys. D: Appl. Phys.* **43**, 095201 (2010).
13. Satyanarayana, S. & Jaluria, Y. A study of laminar buoyant jets discharged at an inclination to the vertical buoyancy force. *Int. J. Heat Mass Transfer.* **25**, 1569-1577 (1982).
